# Otorhinolaryngological profile and surgical intervention in patients with HIV/AIDS

**DOI:** 10.1038/s41598-018-27761-y

**Published:** 2018-08-13

**Authors:** Shiping Bao, Shan Shao

**Affiliations:** Department of Otolaryngology, Head and Neck Surgery, Beijing You’an Hospital, Capital Medical University, Beijing, 100069 China

## Abstract

Little is known about the diagnosis and surgical management of head and neck conditions in patients with HIV/AIDS. This study was conducted to characterize the otorhinolaryngological (ORL) profiles, surgical interventions and outcomes in patients with HIV/AIDS. This retrospective study included patients with HIV/AIDS who underwent head and neck surgeries at You’an Hospital from November 2009 to February 2017. Patients’ ages, ORL diagnoses and surgical interventions for all ORL surgeries were recorded. We identified 57 ORL surgeries in 52 patients during this time. The mean age of the patients was 37.7 ± 12.8 years, with a predominance of male patients (90.4%). The three most common surgical diagnoses were chronic tonsillitis (19.3%), followed by chronic rhinosinusitis (CRS) (14.0%) and vocal polyps (8.8%). The three most common surgeries performed were tonsillectomy (19.3%), endoscopic sinus surgery + radiofrequency ablation of the inferior turbinate (14.0%) and vocal cord polypectomy (8.8%). No mortality occurred in the 30 days after surgery, but 2 patients (3.8%) developed post-operative surgical site infections (SSI). These findings provide information on ORL manifestations and surgical interventions in patients with HIV/AIDS and may assist in the achievement of the most appropriate treatments for this patient population.

## Introduction

Human immunodeficiency virus (HIV) infection/acquired immunodeficiency syndrome (AIDS) is a global pandemic. Since the first case of HIV infection was detected in the early 1980s, the HIV/AIDS epidemic has dramatically spread across the world^1^. Globally, 2.1 million people were estimated to have become newly infected with HIV in 2015. In the same year, an estimated 1.1 million people died of HIV-related illnesses. At the end of 2015, an estimated 36.7 million people were living with HIV^2^. In China, approximately 50,000 patients are newly diagnosed with HIV-1 annually^3^. The increased prevalence of patients with HIV/AIDS has resulted in a greater number of HIV-infected patients visiting otolaryngologists. Approximately 80% of individuals with HIV/AIDS present with various otorhinolaryngological (ORL) conditions^4,5^.

In the early years of the HIV/AIDS epidemic, surgeons were hesitant to perform surgical procedures on these patients due to the higher occurrence of perioperative morbidity and mortality than in non-HIV/AIDS patients^6^. With the advent of highly active anti-retroviral treatment (HAART) in 1990s, HIV has changed from a life-threatening condition with significant morbidity and early mortality to a managed chronic disease that allows patients to lead an active lifestyle with a near-normal life span^7–9^. As the life expectancy of these patients increases, a greater number of patients with HIV/AIDS visit otorhinolaryngologists and become eligible for surgical interventions for conditions that would not previously have been operable^10^.

Therefore, otolaryngologists are experiencing greater challenges in accurately identifying these surgical ORL conditions and providing proper surgical options for HIV-infected patients. However, to date, limited information is available regarding the spectrum of surgical ORL conditions or the availability of surgical interventions for patients with HIV/AIDS. Similarly, little is known about the ORL surgical outcomes in these patients. Based on these considerations, we conducted this study to identify common surgical ORL diagnoses and treatments for patients with HIV/AIDS as a primary objective and to evaluate surgical outcomes in these patients as a secondary objective.

## Results

### Demographics

**Figure 1:**
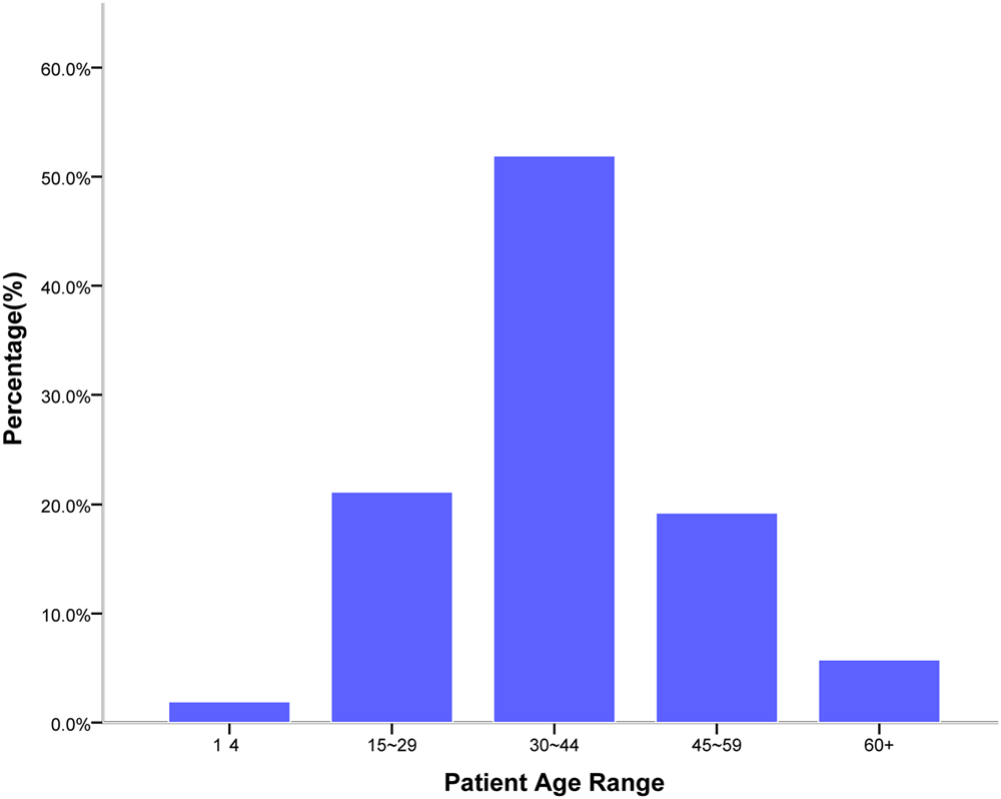
Age range for surgical ORL patients with HIV/AIDS (n = 52).

Of the 52 study participants, 90.4% were male (n = 47) and 9.6% were female (n = 5), with a male to female ratio of 9.4:1. The mean (range) age of the participants was 37.7 ± 12.8 (9–67) years. The most common age group was 30–44 years (Fig. 1).

### HIV Status

The median (range) duration of AIDS was 20.5 (0–240) months, with a mean (range) CD4-positive cell count of 489.8 ± 191.9 (185–915) cells/mm^3^.The CD4-positive cell counts were <200 cells/mm^3^, 200–500 cells/mm^3^, and >500 cells/mm^3^ in 3.8%, 63.5%, and 32.7% of patients, respectively. A total of 67.3% of patients (n = 35) were treated with systemic HAART at the time of surgery. The HIV clinical stage classification was distributed as follows: stage I (50%), II (44.2%), and IV (5.8%). Preoperative viral load was available in 38(73.1%) cases, of which 26 cases were under the level of viral detection <40 copies per milliliter. None of these HIV/AIDS patients were co-infected with active pulmonary tuberculosis (TB) or extrapulmonary TB in the head and neck regions.

### Spectrum of Surgical ORL Diagnoses

**Figure 2:**
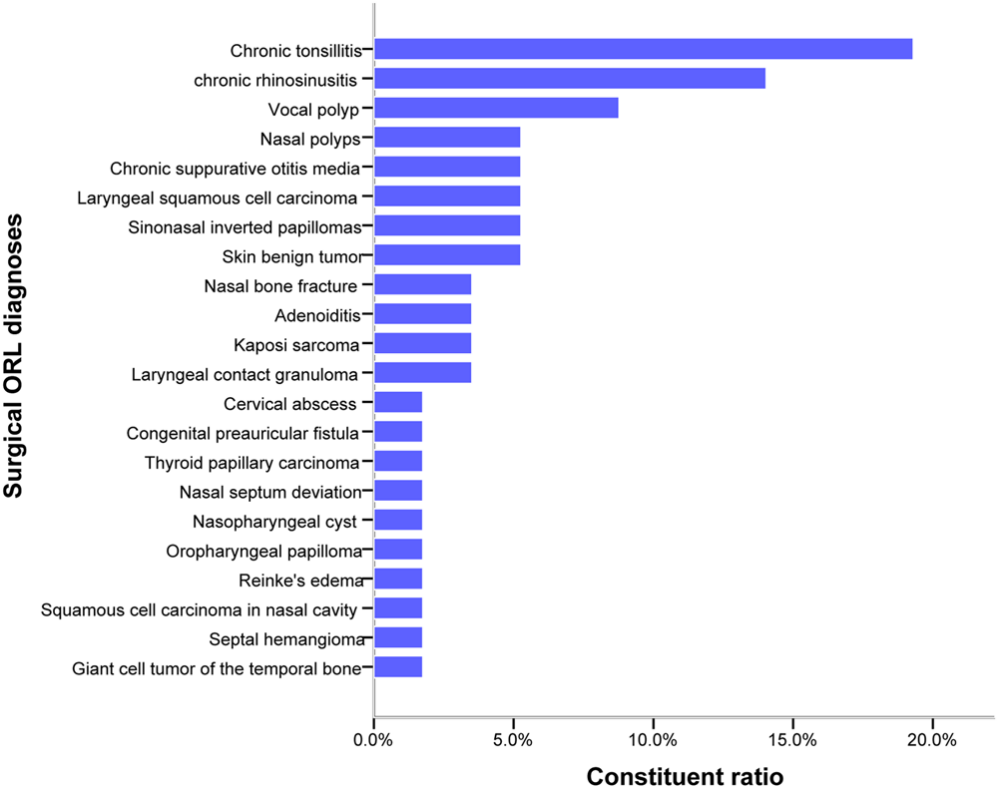
Spectrum of surgical ORL pathological diagnoses in patients with HIV/AIDS.

Fifty-two patients were identified and 57 operations were included in our study. The spectrum of diagnoses encountered is shown in Fig. 2, and the overall most common diagnosis was chronic tonsillitis (19.3%), followed by CRS (14.0%) and vocal polyps (8.8%).

**Figure 3:**
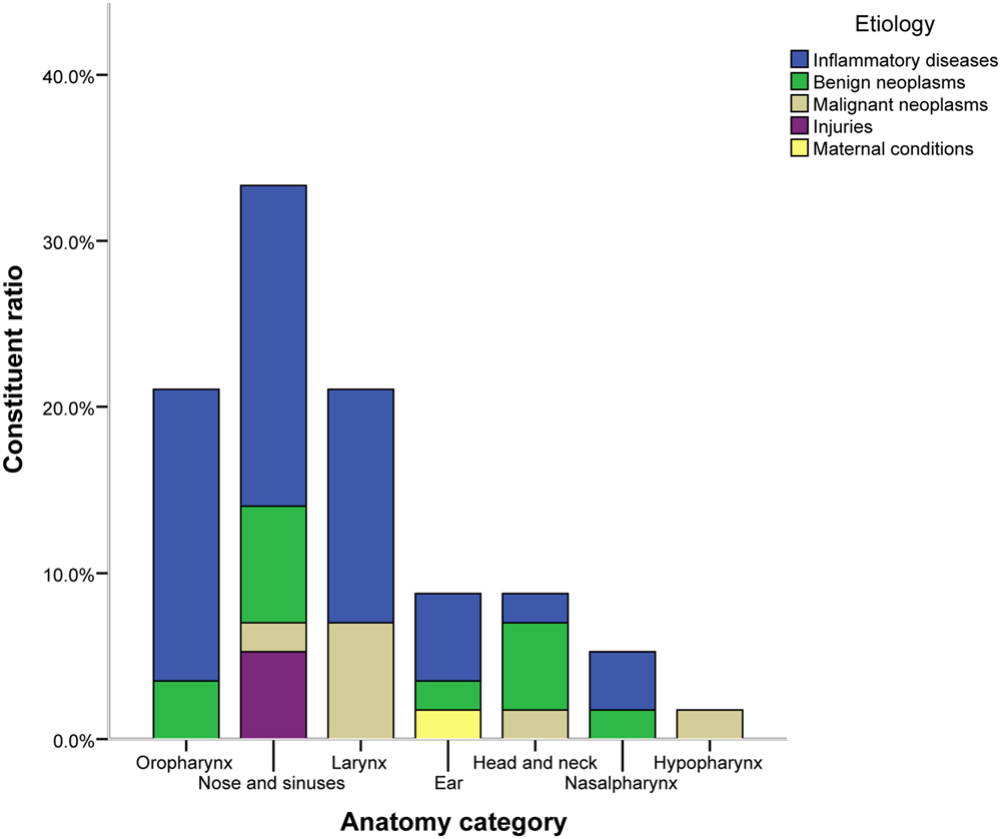
Distribution of etiology category classified by anatomic location.

From an anatomical perspective, nasal and sinus diseases were the most common (33.3%), followed by conditions of the oropharynx (21.1%) and larynx (21.1%). From an etiological classification perspective, inflammatory diseases accounted for 61.4% (n = 35) of cases, and malignant neoplasms accounted for 12.3% (n = 7) of cases. The most common diagnoses by anatomical site were CRS (42.1%) in the nose and sinuses, tonsillitis (91.7%) in the oropharynx, vocal polyps (41.7%) in the larynx, CSOM (60.0%) in the ears, and benign neoplasms (60.0%) in the head and neck, as shown in Fig. 3.

Of note, one patient had a painful neck mass for five months with increasing of the size. The results of computed tomography showed a highly susceptible presentation of cervical tubercular abscess. Chest X ray was normal, mantoux tuberculin skin test (TST) and interferon-γ release assays (IGRA) were negative. Histopathological results finally confirmed the diagnosis of an infectious abscess.

### Surgical Interventions

**Figure 4:**
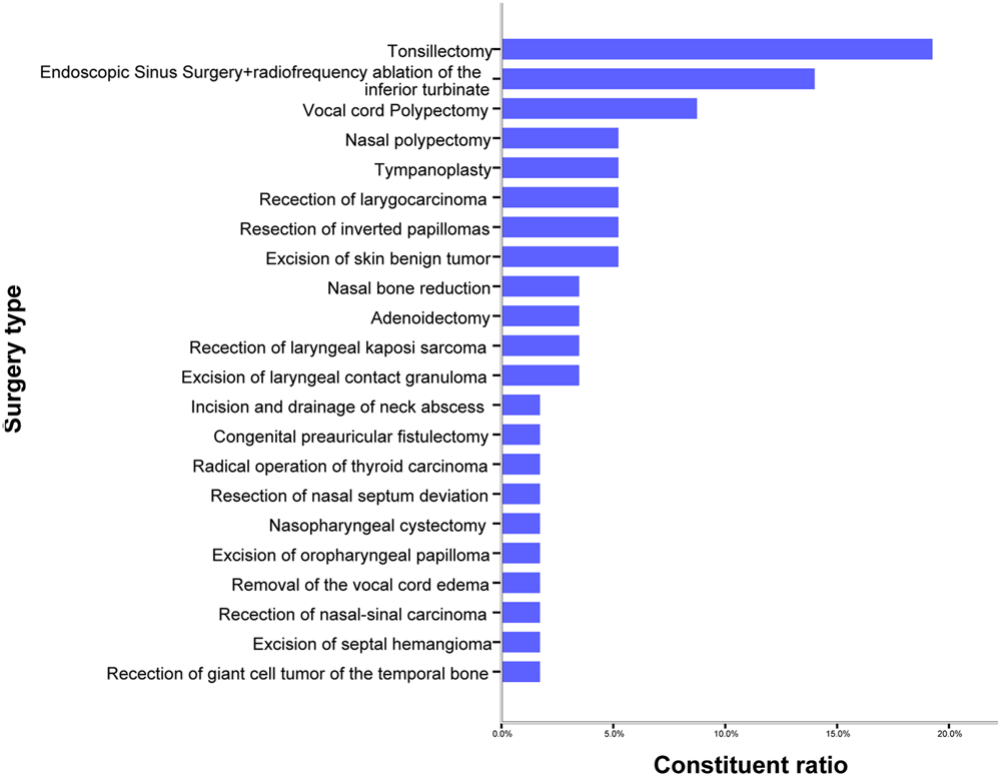
ORL surgical procedures in patients with HIV/AIDS.

The surgical procedures performed are described in Fig. 4, and the most common were tonsillectomy (19.3%), endoscopic sinus surgery + radiofrequency ablation of the inferior turbinate (14.0%) and vocal cord polypectomy (8.8%). Of the 57 surgeries, 50 (87.7%) were elective surgeries, 7(12.3%) were semi-elective surgeries. Majority of the 57 surgeries were minimally invasive endoscopic operations (52, 91.2%), and only 5 (8.8%) open surgeries were performed.

### Surgical outcomes

No mortality occurred in the 30 days after surgery in our study. Of the 52 patients studied, 2 (3.8%) developed surgical wound infections. One case involved a superficial incisional SSI following incision and drainage of a neck abscess with a CD4+ T cell count of 396 cells/mm^3^, and the other case involved nasal discharge following endoscopic nasal sinus surgery, with a CD4+ T cell count of 370 cells/mm^3^. These infections resolved after debridement and prolonged antibiotic treatment.

## Discussion

The earliest reports of surgical procedures in patients with HIV/AIDS reported complication rates as high as 40% and mortality ranging from 55% to 70%^11^. Surgeons have been hesitant to perform surgical procedures on patients with HIV/AIDS because of the higher perioperative morbidity and mortality in these patients than in non-HIV/AIDS patients, and most clinicians recommend medical management or conservative approaches for conditions that are usually treated with surgery^11^. With the development of HAART and an improved quality of life, combined with advances in surgical methods and instruments, an increasing number of patients with HIV/AIDS are likely to develop diseases that require surgical intervention, and a corresponding increase in the surgical burden among HIV/AIDS patients is predicted^3^. However, the spectrum of surgical ORL diagnoses and outcomes of surgical interventions have been poorly studied in patients with HIV/AIDS, particularly in the era of HAART.

In Europe, Africa, and America, patients with HIV/AIDS generally receive surgery in general hospitals, whereas in China, patients with HIV/AIDS are referred to a special hospital for infectious diseases to receive treatment^3^. You’an Hospital, a research-oriented hospital located in Beijing, is the designated hospital for the treatment of patients with HIV/AIDS in China. In the past few years, patients with HIV/AIDS in China have experienced difficulty in receiving ORL surgeries. You’an Hospital began building its own ORL Head and Neck Surgery Department in 2009 to address this problem. In recent years, nearly all patients with HIV/AIDS in China have been referred to and have undergone ORL/head and neck surgeries at our hospital. The surgeons at our hospital have gradually obtained vast experience in treating patients with HIV/AIDS. Our work, which is based on these clinical records, aimed to identify the spectrum of surgical ORL pathological diagnoses and surgical outcomes in patients with HIV/AIDS.

In our study, the most common age group was 30–44 years (42.2%), consistent with the predominance of HIV infection among young people and the age distribution of patients with HIV/AIDS presenting with ORL manifestations^1,3,4,10,12–15^. The male predominance observed in our study was higher than in previous reports of ORL conditions in patients with HIV/AIDS but was consistent with the general HIV-infected population in China, as homosexual men have been the fastest-growing risk group in China’s HIV epidemic in recent years^14,15^.

The spectrum of ORL diagnoses in patients with HIV/AIDS in our study was different from that reported in other developing nations. The top three ORL diagnoses in our study were chronic tonsillitis (19.3%), CRS(14.0%) and vocal polyps(8.8%) respectively. In a prospective study by Tshifularo M *et al*.^4^, adenoid hypertrophy/hyperplasia (AH) (41.6%) was the most prevalent ORL manifestation in South African patients with HIV/AIDS, followed by cervical lymphadenopathy (39%) and CSOM (27%); in India, the most common ORL manifestations were oral candidiasis (20%) and cervical lymphadenopathy (20%)^5^. These differences in the distribution of ORL diagnoses may be attributable to geographical characteristics and differences in the sources of observational data.

After classifying diagnoses by anatomical location into the oropharynx, nose and sinuses, larynx, ear, nasopharynx, hypopharynx, and head and neck, rhinonasal conditions were the most common, followed by oropharynx and larynx conditions. The anatomical location of ORL manifestations varies in published reports from different parts of the world. According to Kirti YK^5^, oropharyngeal manifestations were the most common, similar to previous studies in India^5,16^ and Iran^13^. In contrast to these findings, otological manifestations were more prevalent in South Africa^4^.

We divided the ORL pathological diagnoses in our study into five categories by etiology^17^: injuries, infections, benign neoplasms, malignant neoplasms, and maternal conditions. Infectious conditions were the most common diagnoses and included nearly all ORL regions. The top three infectious diseases in our study were chronic tonsillitis, CRS and chronic suppurative otitis media respectively. The second most common pathological diagnosis was benign neoplasm, including sinonasal inverted papilloma (5.3%), skin benign tumor (5.3%), oropharyngeal papilloma (1.8%), nasopharyngeal cyst (1.8%) and giant cell tumor of the temporal bone (1.8%). Malignant neoplasms included laryngeal squamous cell carcinoma (5.3%), Kaposi sarcoma (KS) (3.5%), thyroid papillary carcinoma (1.8%), and squamous cell carcinoma in nasal cavity (1.8%). Only a few cases were caused by injuries. The condition named congenital preauricular fistula was related to maternal conditions. Our findings provide important and unique data regarding ORL etiological classification and pathological diagnoses for patients with HIV/AIDS from a developing country.

Another key finding was the incidence of non-AIDS-defining cancers (NADCs), which accounted for 8.8% of the total surgical burden among patients with HIV/AIDS in our study. Ziyi Jin, *et al*.^18^ recently reported that NADCs had shown increasing incidence and accounted for a rising proportion of all cancers in this population. Factors including the greater availability of HAART, a high prevalence of co-infections and the higher use of smokeless or smoked tobacco may all contribute to a greater risk of developing NADCs in patients with HIV/AIDS^18,19^. Kaposi sarcoma (KS) is a malignant neoplasm frequently associated with HIV/AIDS; it typically involves the skin, mucosa, and lymphatics and often occurs as a late complication once the CD4 count decreases to <200 cells/mm^3 ^^20^. Notably, in our study, KS was found in adjacent regions at different times in the same patient, who received HAART before surgery, had no detectable viral load and had a CD4-positive cell count greater than 200 cells/mm^3^.

TB and HIV infections are two major public health problems in many regions of the world, particularly in developing countries. With the continued HIV epidemic, extrapulmonary TB now represents about one-fifth of TB cases in the USA^21^. Tuberculous lymphadentitis is the most common presentation of extraplumonary TB, with the neck as a primary target. Of note, none of the patients was co-infected with TB or suffering from head or neck tuberculosis lymphadenitis in our study, although one patient had the initial presentation posing a diagnostic and therapeutic challenge. Previous meta analysis of TB/HIV co-infection in China also reported a lower prevalence of 7.2% (4.2–12.3%)^22^. An explanation could be the selection bias in the study population, because the gender and age distribution in the subjects with severe disease was different from the general population.

HIV/AIDS patients presenting with surgical diseases may be divided into two clinical categories: (a) life-threatening surgical correctable disease and, (b) surgical intervention intended for diagnosis, prophylaxis, or palliation. The consensus is that in the first instance surgical intervention is obligatory but in the second instance alternatives to surgery can be contemplated^23^. In our study, we attempted to perform 57 ORL surgeries in patients with HIV/AIDS, 87.7% were elective surgeries, 12.3% were semi-elective surgeries. Surgical interventions were indicated with benign and malignant neoplasms, infectious diseases failed to conservative treatment, trauma related structural abnormalities. Given that operating on a healthier patient with a higher CD4 count and a lower viral load is advantageous for both patient and surgeon^23,24^, it has been encouraged in our department to postpone elective surgeries with the aim to provide patients with HAART therapy.

Surgical intervention for these ORL conditions varied, and the most common were tonsillectomy (19.2%), endoscopic sinus surgery + radiofrequency ablation of the inferior turbinate (14.0%), and vocal cord polypectomy (8.8%). In a review of 229 cases from a general hospital in Haiti by Kligerman MP *et al*.^25^, tonsillectomy (23.6%) was the most common ORL surgery, which was similar to reports from the United States, where tonsillectomy is the second most common ORL surgery, second only to tympanostomy tube placement. Many diagnoses and surgeries were not recorded in the study by Kligerman and colleagues^25^. For instance, nose, sinus, and ear surgeries were rarely performed, possibly due to a lack of tools and training for endoscopic or microscopic surgery.

This study revealed the safety of ORL surgeries in this high-risk patient group, as no mortality and two SSIs (3.5%) were recorded. The low SSI rate observed in our patients suggests that the risk of SSI is minimal with adequate control of the HIV infection and appropriate perioperative management. In a study of 803 HIV-1-infected patients undergoing surgery in China by Feng TN *et al*.^3^, 313 (38.9%) developed sepsis and 17 patients (2.11%) died within 30 days. Immunocompetent patients undergoing the same surgeries in the same hospital during the same period had a much lower incidence of postoperative sepsis (approximately 3%). SSI following head and neck cancer surgery were reported in up to 10–45% of cases, despite antibiotic prophylaxis. The rate of post-operative complications was relatively low in our study compared with that in previous studies, possibly due to the broad use of minimally invasive surgical techniques, minimal trauma during surgery, accumulation of perioperative experience, improvements in surgical skills and early use of HAART.

Nevertheless, our study has some limitations. First, we were unable to assess HIV-negative patients who underwent ORL surgeries at our hospital. Thus, although we have a strong understanding of common surgical ORL conditions in patients with HIV/AIDS, we have little data on ORL conditions in HIV-negative patients in our department. Second, this study included a small sample. Further prospective analyses on a national scale and with a much larger cohort of patients are needed.

In conclusion, ORL conditions constitute a common yet underserved surgical problem in patients with HIV/AIDS. ORL and head and neck manifestations in patients with HIV/AIDS are common and widespread. ORL surgery in these patients is generally safe, with a relatively low SSI and mortality rate, but patients’ general and ORL health should be optimized preoperatively. Otolaryngologists should be aware of ORL manifestations and surgical treatments to perform more effective evaluations and consider the most appropriate treatments for this group of patients.

## Methods

### Study participants

Records for patients with HIV/AIDS who underwent ORL surgeries between November 2009 and February 2017 at the ORL and Head and Neck Surgery Department of Beijing You’an Hospital (Capital Medical University, Beijing, China) were reviewed. This study was approved by the Ethics Committee of Beijing You’an Hospital. The methods were performed in accordance with the relevant guidelines and regulations approved by the Ethics Committee of Beijing You’an Hospital, Beijing, China. Written informed consent was obtained from the participants prior to data collection.

### Data collection

A thorough medical history was recorded and a physical examination of the ORL and head and neck areas was performed on each patient. The variables of interest, including medical history, physical examination, perioperative records such as routine examinations (complete blood count, routine urinalysis, liver function and biochemical examination, preoperative virus screening, electrocardiogram and chest radiograph), HIV-specific variables (CD4+ lymphocyte count, HIV viral load and HAART use), definitive diagnosis (based on histopathological report), type of surgery and outcomes were retrieved. Patients with HIV/AIDS were also followed for the first 30 days after surgery to detect mortality and surgical site infections (SSI) occurring after discharge. ORL surgeries performed at different sites or at different time points for the same patient were counted as separate cases; hence, the total number of surgical diagnoses exceeded the sample size.

### Definitions

HIV disease status was classified using world health organization (WHO) clinical stage classification^24^. SSI were defined as infections occurring within 30 days of the procedure, according to the CDC parameters^26,27^. TB screen in HIV/AIDS patients were conducted using the WHO-recommended symptom screen (cough, fever, night sweats, weight loss)^28^. Patients with a negative symptom screen were regarded as non-active TB. Patients with positive TB symptom screen or suspected TB patients based on imaging test were undergone further TB diagnostic testing (sputum smears, TST, IGRA and/or histopathological examination).

### Statistical analysis

Data were analyzed using SPSS 22.0 (SPSS, Chicago, IL, USA). The descriptive statistics are presented as means ± standard deviations (SD) when the data were normally distributed. Otherwise, the data are presented as medians (lower and upper bounds of the 95% confidence interval (CI)).

### Data availability

The datasets generated or analysed during the current study are available from the correspondence author on reasonable request.
